# Distinct activation modes of the Relaxin Family Peptide Receptor 2 in response to insulin-like peptide 3 and relaxin

**DOI:** 10.1038/s41598-017-03638-4

**Published:** 2017-06-12

**Authors:** Shoni Bruell, Ashish Sethi, Nicholas Smith, Daniel J. Scott, Mohammed Akhter Hossain, Qing-Ping Wu, Zhan-Yun Guo, Emma J. Petrie, Paul R. Gooley, Ross A. D. Bathgate

**Affiliations:** 1 Department of Biochemistry & Molecular Biology, The University of Melbourne, Victoria, Australia; 2 Bio21 Molecular Science and Biotechnology Institute, The University of Melbourne, Victoria, Australia; 3 Florey Institute of Neuroscience and Mental Health, The University of Melbourne, Victoria, Australia; 4 School of Chemistry, The University of Melbourne, Victoria, Australia; 5 Institute of Protein Research, College of Life Sciences, and Technology, Tongji University, Shanghai, China

## Abstract

Relaxin family peptide receptor 2 (RXFP2) is a GPCR known for its role in reproductive function. It is structurally related to the human relaxin receptor RXFP1 and can be activated by human gene-2 (H2) relaxin as well as its cognate ligand insulin-like peptide 3 (INSL3). Both receptors possess an N-terminal low-density lipoprotein type a (LDLa) module that is necessary for activation and is joined to a leucine-rich repeat domain by a linker. This linker has been shown to be important for H2 relaxin binding and activation of RXFP1 and herein we investigate the role of the equivalent region of RXFP2. We demonstrate that the linker’s highly-conserved N-terminal region is essential for activation of RXFP2 in response to both ligands. In contrast, the linker is necessary for H2 relaxin, but not INSL3, binding. Our results highlight the distinct mechanism by which INSL3 activates RXFP2 whereby ligand binding mediates reorientation of the LDLa module by the linker region to activate the RXFP2 transmembrane domains in conjunction with the INSL3 A-chain. In contrast, relaxin activation of RXFP2 involves a more RXFP1-like mechanism involving binding to the LDLa-linker, reorientation of the LDLa module and activation of the transmembrane domains by the LDLa alone.

## Introduction

Relaxin and insulin-like peptide 3 (INSL3) are two members of the insulin superfamily of peptides that are best known for their roles in the reproductive tract of females and males respectively^1^. Relaxin is a pregnancy hormone with roles that differ between species, but common mechanistic actions include tissue remodelling as well as having cardiovascular effects such as vasodilation and organ protection^1–3^. The human relaxin peptide, human gene-2 (H2) relaxin, has attracted recent attention due to its successful passage through three phases of clinical trials for acute heart failure where the drug significantly reduced mortality in groups receiving recombinant H2 relaxin (serelaxin) compared to placebo^4^. The precise mechanism by which relaxin functions in heart failure is not known but is thought to involve numerous positive actions on vessels and organs^5^. INSL3 is predominantly produced in testicular Leydig cells^6^ and plays a vital role in testicular descent in many species^2, 7^. In females INSL3 is found at lower levels than in males, but it is detectable in serum and is largely produced in the theca interna cells of ovarian antral follicles in humans^8^. The role of ovarian INSL3 in humans is currently unknown however animal studies have demonstrated putative roles for thecal derived INSL3 in germ cell survival^9^ and follicular androgen production^10^. In addition to its reproductive roles, INSL3 has been implicated in bone metabolism in males where low levels are associated with an increased risk of osteopenia and osteoporosis^11^. Both hormones are also overexpressed in a number of human cancers^12–16^.

**Figure 1:**
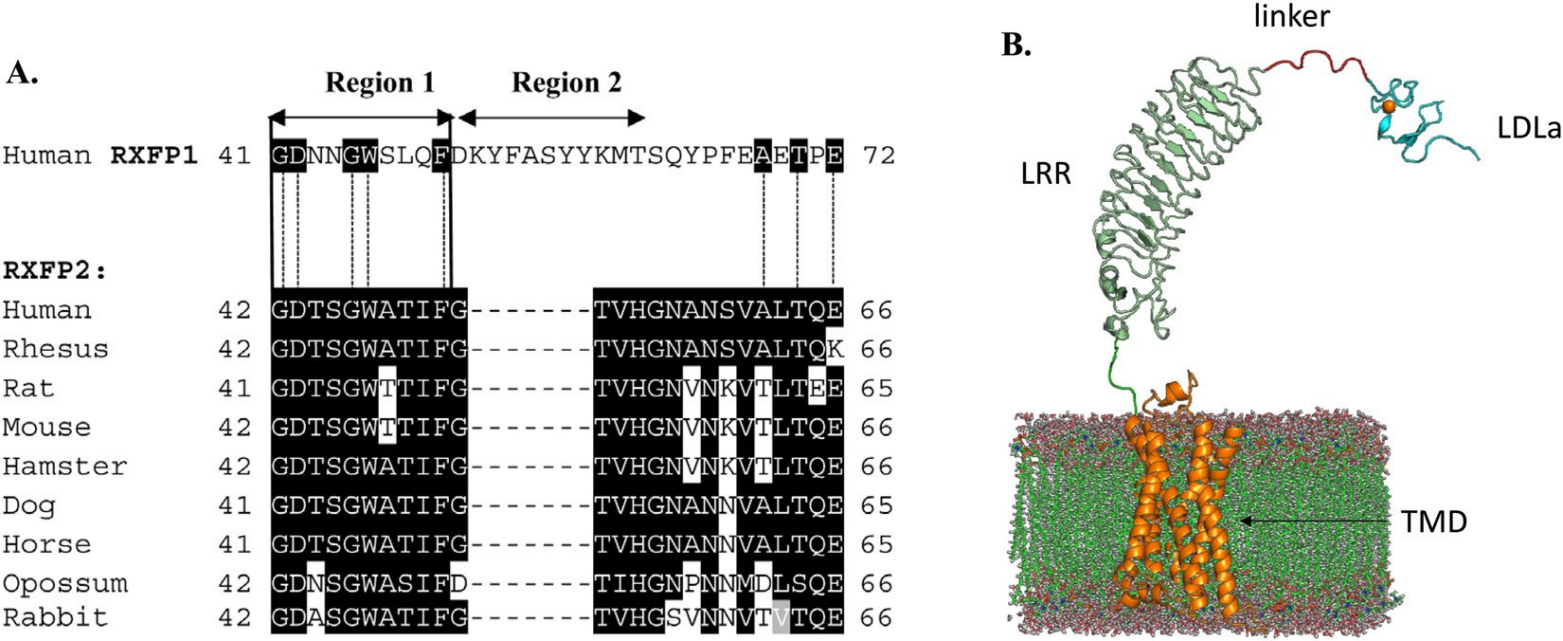
(**A**) Sequence alignment of the 25-residue RXFP2 linker from mammalian species shown with the human RXFP1 linker, which is 32 residues long. Residues GDxxGWxxxF comprise region 1, thought to be important for activation, and region 2 is the proposed relaxin-binding site. Region 2 is absent from the RXFP2 linker. Species shown are human, rhesus (*Macaca mulatta*), rat (*Rattus norvegicus*), mouse (*Mus musculus*), chinese hamster (*Cricetulus griseus*), dog (*Canis lupus*), horse (*Equus caballus*), opossum (*Monodelphis domestica*) and rabbit (*Oryctolagus cuniculus*). Sequences are from UniprotKB. (**B**) Putative structure of RXFP2 showing the distinct protein domains, the transmembrane domain (TMD) shown in orange, leucine-rich repeats (LRR) in green, linker in purple and LDLa module in blue. Calcium ion is an orange sphere.

The peptides are structurally similar to insulin, consisting of an A- and a B-chain that are stabilized by two inter-chain and one intra A-chain disulphide bonds^17, 18^. They act via their respective receptors, the relaxin family peptide receptors RXFP1 and RXFP2, which are G-protein coupled receptors (GPCR) and contain the seven transmembrane alpha-helices typical of this group. However, RXFP1 and RXFP2 are unique in that they also contain a large ectodomain consisting of a series of 10 leucine-rich repeats (LRRs) and an N-terminal low-density lipoprotein type a (LDLa) module (Fig. 1B). This domain structure puts them in the LRR-containing family of GPCRs (LGR) but they make up their own classification within this group, forming class C of the LGRs, as they are the only known mammalian GPCRs to contain an LDLa module. The mechanism by which they bind and are activated by their ligands is therefore an unusual paradigm in GPCR functioning, and it involves a complex interplay between the various receptor and ligand domains and regions. Structural and interaction characterization is necessary in the search for small molecule agonists of RXFP1 which may have improved drug-like qualities compared to the native ligand^19^. Investigation of the similar but not identical functioning of the related receptor RXFP2 is valuable as a comparison to RXFP1 and in its own right as a potential future therapeutic target for conditions including cryptorchidism, osteoporosis and cancer. Additionally H2 relaxin is able to bind and activate RXFP2 with nanomolar affinity^20^ and understanding of this interaction is important in the context of therapeutic development.

Current knowledge holds that both receptors bind their ligand via a high-affinity interaction between the LRRs and the B-chain of the peptide^21, 22^. Importantly, both receptors absolutely require the presence of the LDLa module for signalling to occur^23^. This module is thought to interact with the extracellular loops (EL) of the transmembrane domain (TMD) of RXFP1^24^, thus making the LDLa module the true ligand and uncovering an interesting archetype in GPCR signalling. Other examples of GPCRs making use of tethered ligands include the protease-activated receptors (PARs), which are first cleaved to expose the N-terminal agonist that interacts with the receptor ELs^25, 26^. In the case of RXFP1 and RXFP2 however, it is the binding of the peptide hormone that allows the LDLa to come into contact with the ELs and bring about the final active receptor conformation. The LRRs and LDLa module are connected to each other by a stretch of linking residues termed the linker, and this region differs in length and sequence between the two receptors (Fig. 1A).

We recently showed that the RXFP1 linker is involved in ligand binding and contains a transient helical structure that is stabilized in the presence of relaxin^27^. We additionally found that the residues immediately adjacent to the LDLa module are extremely important for activation, since mutation of Asp42, Gly45 and Trp46 to Alanine leads to vastly reduced signalling. Nuclear magnetic resonance (NMR) studies on a recombinant protein consisting of the RXFP1 LDLa module and linker in titration experiments with a protein displaying RXFP1 ELs revealed that they may be the point of interaction between the linker and EL2. Our current model of ligand-mediated activation therefore suggests that binding of H2 relaxin to the linker stabilizes a helix which then positions residues of the LDLa module and linker to interact with the TMD to activate the receptor.

In this study, we use similar methods to establish the role of the RXFP2 linker, which contains the conserved GDxxGW (where x is any residue) found in RXFP1, but lacks the proposed relaxin binding site (Region 2; Fig. 1A). It should be noted that relaxin is capable of activating RXFP2, albeit with lower potency than INSL3, while the converse is not true, as INSL3 is an extremely poor activator of RXFP1^28^. Mutants were made in the linker region of full-length RXFP2 and tested for INSL3 and H2 relaxin binding and activation. Parallel studies assessed ligand interaction with a soluble RXFP2 LDLa-linker protein using NMR. Our results demonstrate that INSL3 does not bind to the RXFP2 linker but the GDxxGW is absolutely required for activation. In contrast H2 relaxin weakly interacts with the linker and also requires the GDxxGW motif for activation.

## Results

Our previous work on the RXFP1 linker showed that this domain contains two distinct regions involved in activation (Region 1, Fig. 1A) and binding (Region 2, Fig. 1A) respectively^27^. Alignment of the human RXFP1 linker sequence to that of human RXFP2 and other mammalian RXFP2 sequences highlights that the linker is seven residues shorter and that region 2 is missing in all RXFP2 sequences (Fig. 1A). In contrast, region 1 shows high conservation within mammalian RXFP2 sequences and has 100% conservation of the critical RXFP1 D42, G45, W46 and F50 residues (Fig. 1A) which we demonstrated are essential for receptor activity^27^. We therefore explored the role of the putative region 1 in the RXFP2 linker by mutating to alanine each of the first six residues, GDTSGW, as well as Phe51. Mutants of the full-length receptor were transiently transfected into HEK293T cells and tested for both INSL3 and relaxin binding. Receptor activation in response to either ligand was tested by measurement of cAMP activity the main signalling pathway activated by RXFP1 and RXFP2 in HEK-293T cells.

### Role of the RXFP2-linker in INSL3 binding and activation

**Figure 2:**
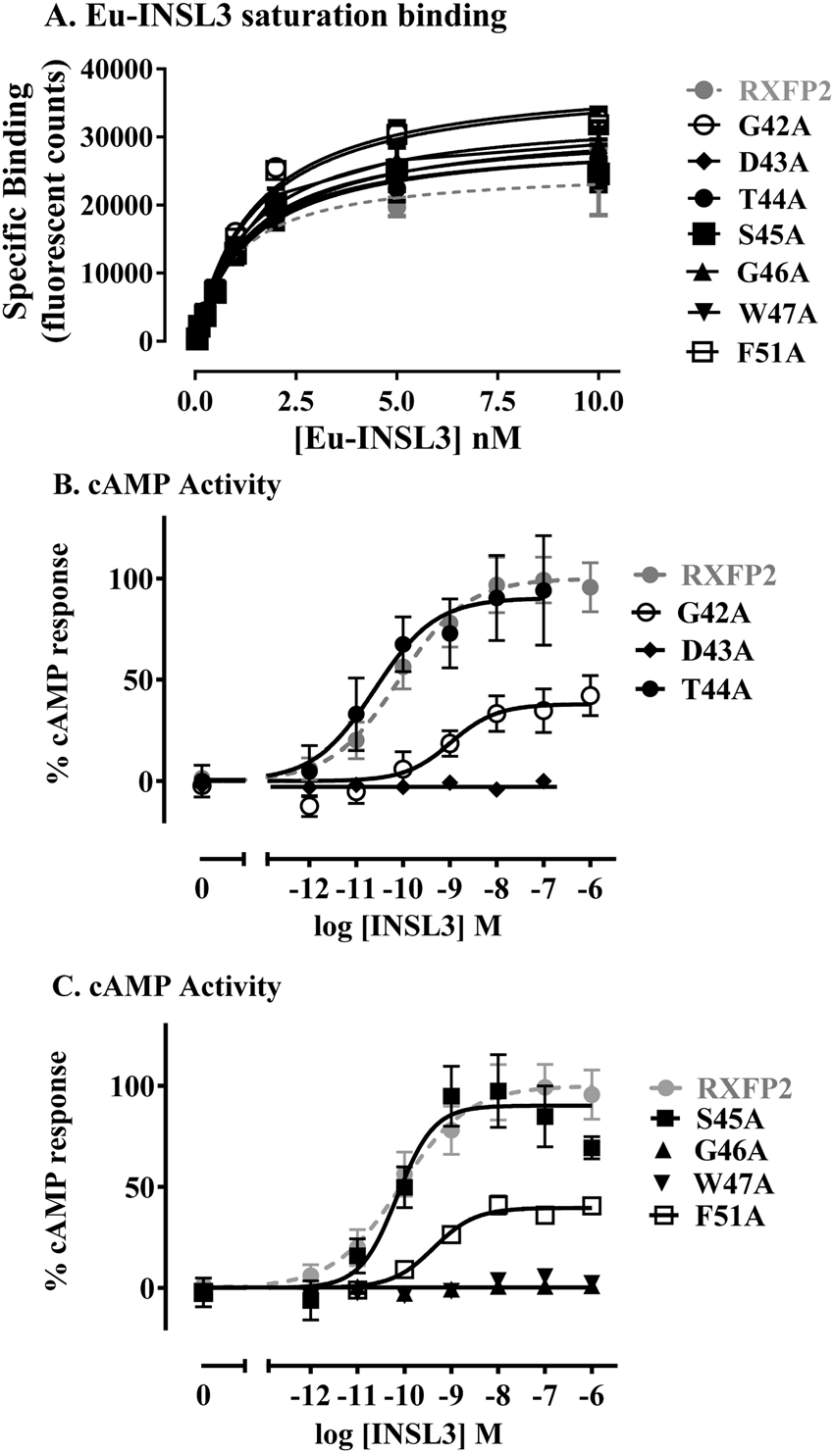
(**A**) Eu-INSL3 saturation binding of RXFP2 mutant receptors compared to wild-type RXFP2. (**B**,**C**) INSL3-induced cAMP response of RXFP2 mutant receptors compared to wild-type RXFP2. Data is presented as mean ± SEM of triplicate determinations from at least three independent experiments.

The mutants were first tested for their ability to bind INSL3 using Eu-INSL3 saturation whole cell binding assays (Fig. 2A, Table 1). The affinity of Eu-INSL3 binding to human RXFP2 (1 nM, Table 1) was similar to what we have previously published using this ligand^29^. Importantly, there was no significant difference in the affinity of Eu-INSL3 binding to any of the mutant receptors and in addition, there were no differences in the B_max_ values indicating that they were all expressed at the cell surface at an equivalent level (Fig. 2A, Table 1).

**Table 1:** Activity data for RXFP2 mutants compared to wild-type RXFP2.

Construct	Eu-INSL3 binding		Eu-H2 relaxin binding		INSL3 activation		H2 Relaxin activation	
	*B*_max_	*K*_d_	*B*_max_	*K*_d_	*E*_max_	pEC_50_	*E*_max_†	pEC_50_
RXFP2	23675 ± 5961 (3)	1.07 ± 0.22 (3)	19543 ± 3673 (3)	9.96 ± 0.62 (3)	100 (6)	9.99 ± 0.40 (6)	100 (6)	8.45 ± 0.15 (6)
G42A	35678 ± 7441 (3)	1.93 ± 0.69 (3)	ND	ND	39.47 ± 12.90 (3)*	8.98 ± 0.45 (3)	75.21 ± 12.02 (3)	7.98 ± 0.55 (3)
D43A	25734 ± 10412 (3)	1.18 ± 0.15 (3)	No binding (3)		No activity (3)		No activity (3)	
T44A	32817 ± 4006 (3)	2.11 ± 0.63 (3)	ND	ND	98.66 ± 29.83 (4)	10.50 ± 0.44 (4)	53.02 ± 5.55 (3)*	8.63 ± 0.40 (3)
S45A	26897 ± 5303 (3)	1.05 ± 0.20 (3)	ND	ND	92.15 ± 21.82 (3)	10.14 ± 0.20 (3)	100 ± 7.23 (4)	9.03 ± 0.13 (4)
G46A	34363 ± 2825 (3)	1.51 ± 0.43 (3)	No binding (3)		No activity (3)		No activity (3)	
W47A	40744 ± 10281 (3)	1.50 ± 0.41(3)	No binding (3)		No activity (3)		No activity (3)	
F51A	39612 ± 5290 (3)	1.55 ± 0.13 (3)	No binding (3)		39.76 ± 3.69 (3)*	9.36 ± 0.09 (3)	18.78 ± 5.0 (3)**	< 6 (3)

*Indicates p < 0.05, **indicates p ≤ 0.01, ***indicates p ≤ 0.001. *E*_max_: maximal cAMP response, % maximal RXFP2 activity; pEC_50_: H2/INSL3 potency of RXFP2 receptor; *K*_d_: dissociation constant for relaxin or INSL3 binding; *B*_max_: maximal binding; ND: not done. Number of individual assays performed in parentheses; ^†^Max value based on 1 µM activation.

In contrast, there were marked effects of the mutations on the ability of the mutant receptors to signal in response to INSL3. Mutants G42A, D43A, G46A, W47A and F51A all showed significantly reduced INSL3-induced cAMP activity, in particular mutants D43A, G46A and W47A showed no response up to concentrations of 1 µM INSL3 (Table 1, Fig. 2B,C). G42A and F51A showed no change in INSL3 potency but the efficacy in response to INSL3 was reduced to around 40% of the maximum response seen by the wild-type receptor. By comparison mutation of Thr44 or Ser45 to alanine had no effect on INSL3 stimulated cAMP activation.

### Role of the RXFP2-linker in H2 relaxin binding and activation

**Figure 3:**
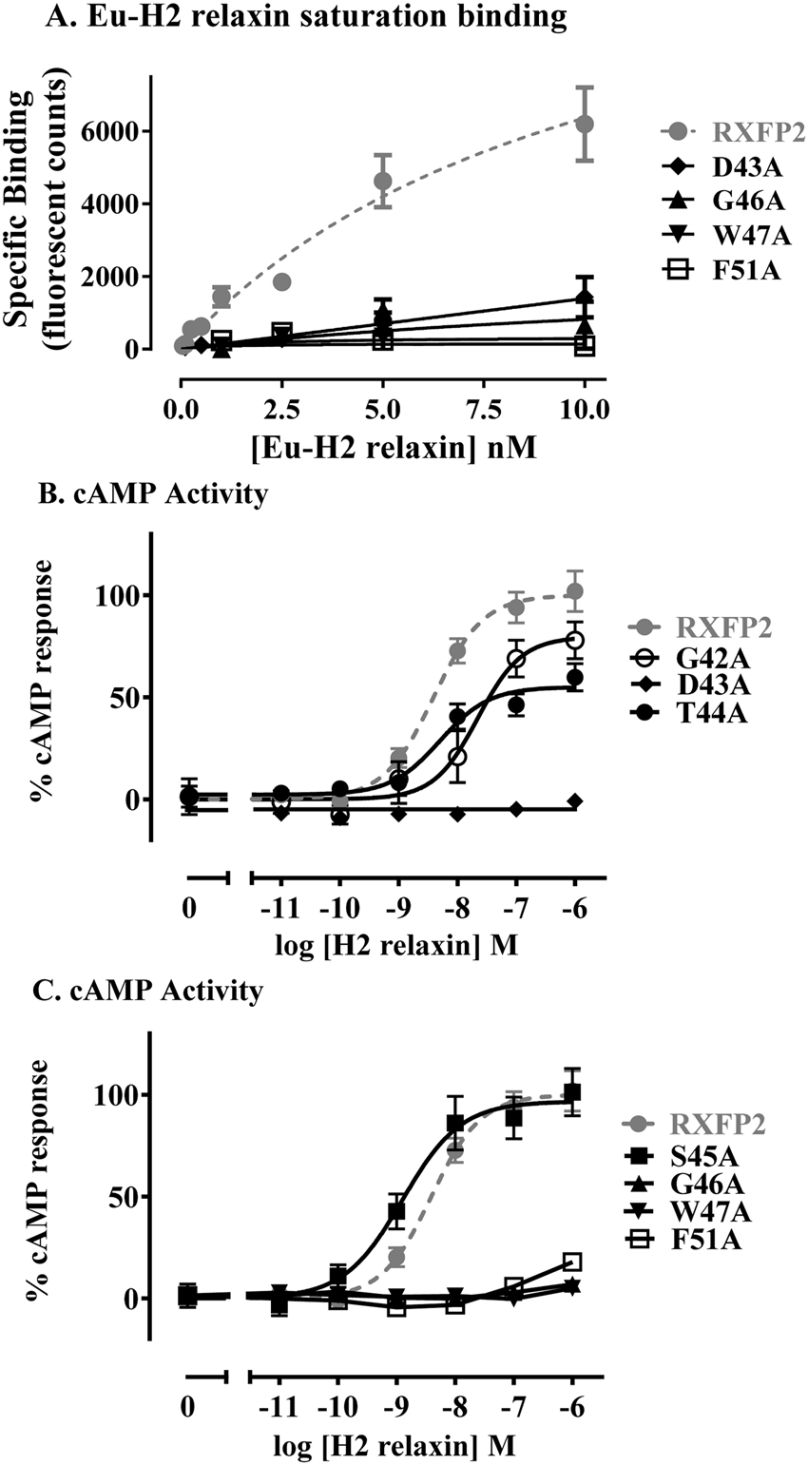
(**A**) Eu-H2 relaxin saturation binding of RXFP2 mutant receptors compared to wild-type RXFP2. (**B**,**C**) H2 relaxin-induced cAMP response of RXFP2 mutant receptors compared to wild-type RXFP2. Data is presented as mean ± SEM of triplicate determinations from at least three independent experiments.

Saturation binding assays to test mutant binding to relaxin using Eu-H2 relaxin showed strikingly different phenotypes to those performed using Eu-INSL3. Wild-type RXFP2 bound relaxin with lower affinity than INSL3 as previously reported in competition binding assays^28^ (Table 1, Fig. 3A). However, no specific binding was seen for any of the linker mutants tested, namely D43A, G46A, W47A and F51A up to a concentration of 10 nM Eu-H2 relaxin (Table 1, Fig. 3A).

Signalling in response to relaxin demonstrated some similarities but also distinct differences to that seen upon INSL3 testing. Comparable to INSL3 responses, no activity was observed from mutants D43A, G46A or W47A in response to relaxin concentrations up to 1 µM. T44A and S45A both demonstrated no changes in relaxin potency however the efficacy of T44A in response to relaxin dropped by around half. The G42A mutant showed a reduction in both efficacy and potency in response to relaxin but these decreases were not as profound as seen in response to INSL3 (Table 1, Fig. 3B). Finally, while the F51A mutant demonstrated reduced INSL3 efficacy and potency it was virtually unresponsive to relaxin with only a small response at 1 µM concentration (Table 1, Fig. 3C). These results parallel what was seen for equivalent RXFP1 linker mutants in response to relaxin^27^, and further highlight the different binding modes utilised by the two related peptides.

### Production of B5-29 [B-K 9 R,A-K9/17 R] (truncated) H2 relaxin

Previous studies using the native H2 relaxin peptide have demonstrated that the peptide has a propensity to dimerize at high concentrations^30^. As this study demonstrated that dimerization involves the N-terminus of the B-chain and it is known that this region can be removed without affecting relaxin activity at both RXFP1 and RXFP2^31^ we decided to produce a B-chain truncated H2 relaxin peptide for NMR studies. A fully active H2 relaxin analog, in which three Lys residues of human relaxin-2 were all replaced by Arg residues and is easily produced and purified in a *Pichia pastoris* expression system^32^, was used as the template to generate the truncated relaxin peptide. To remove the B-chain N-terminal fragment that is responsible for relaxin dimerization a B-M5S mutation was introduced to the template so that the mutant B-chain N-terminal tetrapeptide (Asp-Ser-Trp-Met) would be conveniently removed by chemical cleavage at the carboxyl side of the unique Met residue by CNBr. After overexpression, purification, and *in vitro* maturation truncated relaxin was obtained with correct molecular mass (measured value 5484.0; theoretical value 5483.4) and high yield (~5 mg mature peptide from one litre of culture broth). Truncated relaxin demonstrated equivalent potency to native H2 relaxin in cAMP activity assays in HEK-293T cells expressing RXFP1 or RXFP2 (Supp. Fig. 1). Similar to relaxin that is amidated at the C-termini^33^, truncated relaxin has markedly improved NMR properties. A comparison of 2D ^1^H NOESY and TOCSY spectra (Supp. Fig. 2) of H2 relaxin and truncated relaxin at 500 µM showed more cross-peaks in the latter spectra supporting that truncated relaxin, like amidated relaxin, is monomeric.

### Mapping the H2 relaxin-binding site on RXFP2(_1–65_)

**Figure 4:**
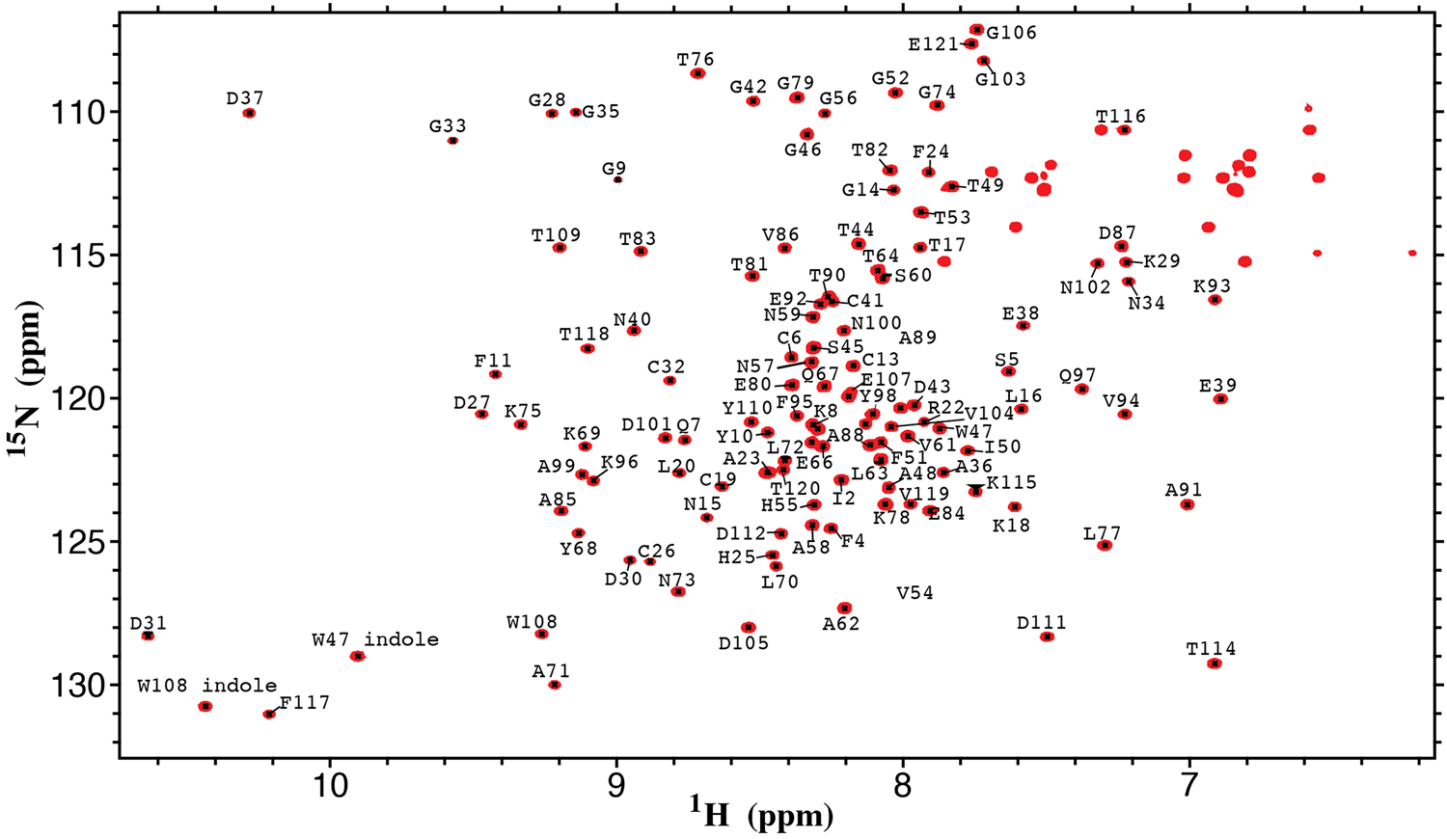
Full ^1^HN ^15^N HSQC spectrum of RXFP2_(1–65)_ P4F with residue assignments. Unmarked resonances in the upper right corner belong to Asn and Gln sidechains and have not been assigned. Residues I2 to E66 correspond to the LDLa-linker while residues Q67 to E121 belong to the GB1 module.

When studying the interactions between relaxin and the RXFP1 LDLa-linker we used a recombinant construct consisting of the LDLa module and the 32 residues linking the module and the LRR domain. In this way we were able to analyse the low affinity interaction between relaxin and the LDLa-linker using NMR experiments^27^. Following a similar rationale for the RXFP2 region, we designed a construct consisting of the LDLa module and the first 25 residues of its linker. In this case however, the protein was fused at its C-terminus to GB1, a thermostabilized version of the B1 immunoglobulin binding domain of streptococcal protein G (GB1), which acts as a solubility and stability enhancement tag^34^. This construct is designated RXFP2_(1–65)_ and it was recombinantly expressed and purified. 2D ^1^H-^15^N HSQC spectra of this construct, as well as the equivalent construct lacking the GB1 domain, show that the RXFP2 portion behaves identically both in the absence and presence of GB1. Nevertheless, a distinct heterogeneity was seen in the spectra, most easily observed for the indole resonance of W47 (Supp. Fig. 3). This heterogeneity was suspected to be due to the presence of cis-trans isomerization of Pro4 resulting in splitting of resonances of residues nearby in the structure. We therefore mutated Pro4 to phenylalanine and this gave us a homogeneous spectrum which was used in further experiments (Fig. 4 and Supp. Fig. 3). Importantly, mutation of this residue was tested for signalling in full-length receptor (Supp. Fig. 4) and its activity was uncompromised in response to both relaxin and INSL3. Expression and purification of protein with ^13^C,^15^N-labeling enabled the assignment of the backbone resonances using standard triple-resonance methodology (Fig. 4).

**Figure 5:**
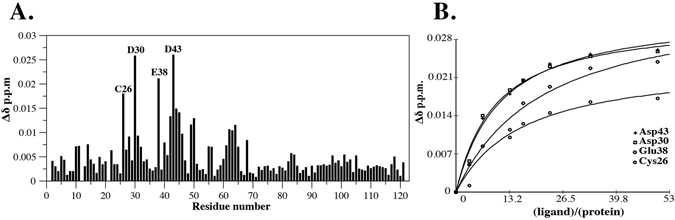
(**A**) Plot of the change in average ^1^HN and ^15^N chemical shifts following titration of ^15^N-RXFP1_(1–72)_ P4F with 50 equivalents of truncated relaxin. (**B**) Single-site saturation binding curves (K_d_ = 330 ± 10 μM) for the resonances that show the largest chemical shift changes and remained resolved throughout the titration.

A 2D ^1^H-^15^N HSQC monitored titration was then carried out on ^15^N-labelled RXFP2_(1–65)_ P4F with increasing concentrations of truncated relaxin. Significant chemical shift changes were observed throughout both the linker and LDLa module (Fig. 5). Residues assigned to the GB1 module (residues Q67 to E121) do not show any change even at the highest concentrations of truncated relaxin, indicating that the interaction is specific to the RXFP2 portion of the construct. Residues Asp30 and Glu38 that are involved in calcium binding^35^ were among the most altered as well as Cys26 and Asp43, the latter being a linker residue that showed great perturbations in both relaxin and INSL3 signalling as well as relaxin binding. Since the proposed relaxin binding region of RXFP1 is not present in the RXFP2 linker (Fig. 1A), it stands to reason that any binding site should be distinct, and similarly that the affinity of LDLa and/or linker should be lower, which is indeed the case. Plotting the chemical shift changes of residues that showed significant shifts on addition of increasing concentrations of truncated relaxin gives a K_d_ of around 330 µM (Fig. 5B). For comparison, we titrated RXFP1 LDLa-linker with truncated relaxin and show the same site of interaction to that observed for relaxin^27^ (Supp Fig. 5). However, this interaction with truncated relaxin is more potent showing a K_d_ of 90 µM, which also is more than three-fold stronger than that observed for the RXFP2_(1–65)_.

### LDLa and linker interactions with the TMD exoloops

We have previously demonstrated that chimeric RXFP1 receptors with an RXFP2 LDLa module can signal in response to relaxin, although the presence of the native linker seems essential to this activity^36^. In order to explore the interaction of the RXFP2 LDLa module and the LDLa-LRR linker with the TMD extracellular loops (EL) we used the same approach that we employed within the RXFP1 system^27^. We designed a soluble scaffold based on GB1 onto which was grafted the partial 1^st^ and entire 2^nd^ exoloops of RXFP1 or RXFP2. Designated ssRXFP1 and ssRXFP2, the constructs contain a disulphide bridge between loops 1 and 2 that is essential for correct receptor structure and function^37^. Characterisation of the presence of structure in ssRXFP2 was done using circular dichroism (CD) spectrophotometry, which showed 23.4% helix, 34.5% strand, 10.7% turns and 21.8% unordered (Supp. Fig. 6). These are similar values to those seen for thermostabilised GB1 (tGB1) previously^24^, although the construct has significantly less helical structure (tGB1 value 35.5%) and a greater percentage of strand (tGB1 value 14.7%) according to our analysis.

**Figure 6:**
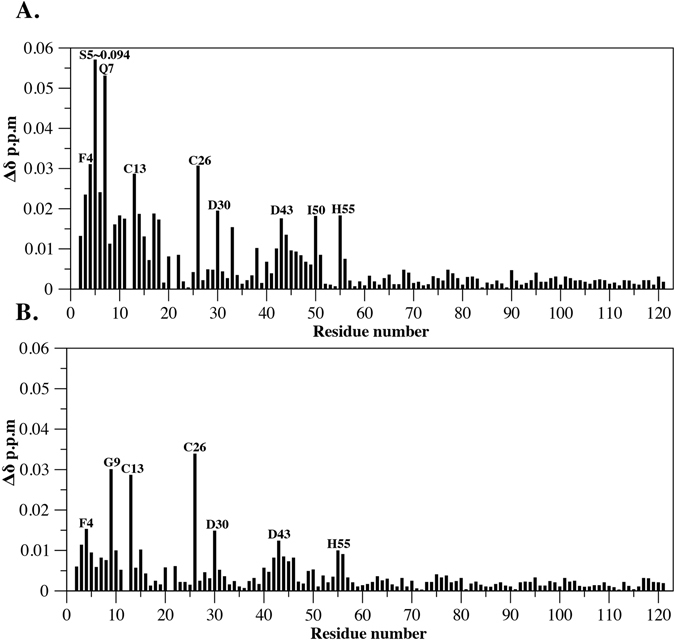
Plot of the change in average ^1^HN and ^15^N chemical shifts following titration of ^15^N-RXFP2_(1–65)_ P4F with 20 equivalents of (**A**) ssRXFP1 and (**B**) ssRXFP2.

We performed titrations of ^15^N-labelled RXFP2_(1–65)_ P4F with increasing concentrations of both ssRXFP1 and ssRXFP2. Interestingly, in response to both scaffold proteins an interaction was seen most strongly around the N-terminal of the LDLa module. While small chemical shift changes occurred in the linker, residues Cys13 and Cys26 moved significantly in both experiments, as did Ser5 and Gln7 in response to ssRXFP1 (Fig. 6A), and Gly9 after addition of ssRXFP2 (Fig. 6B). To show that the interaction is dependent on the conformation of EL2 we titrated a version of ssRXFP1 in which the cysteine residues were mutated to serine such that the loop disulphide bond would not form and the conformation of the EL2 is therefore not restricted^24^. In this experiment no chemical shift changes were observed supporting dependence on the conformation of EL2 for LDLa-linker interactions (Supp. Fig. 7).

## Discussion

RXFP1 and RXFP2 are two unique GPCRs that play diverse and important roles within female and male reproductive systems as well as other fundamental physiological processes (reviewed in refs 38 and 39). Our previous studies have demonstrated that both receptors require the unique LDLa module to signal in response to their ligands^23^ and suggested that it acts as a tethered ligand^40^. Our more recent work on RXFP1 showed that the linker domain flanking the LDLa module interacts with H2 relaxin and that this interaction stabilizes a helical structure within the linker allowing the LDLa module and linker residues to interact with the TMD^27^. This highlighted the potential mechanism by which ligand binding can direct LDLa mediated receptor activation. In this study, we have investigated the role of the equivalent linker domain in RXFP2 to elucidate if the activation mechanism is similar.

Unlike RXFP1, RXFP2 can interact with both relaxin and INSL3, although we have previously shown that the two ligands bind to the primary binding site in the LRRs in a distinct manner^41^. This study focussed on the linker between the LDLa module and LRRs, and sought to discover whether this region was equally important in both receptors. While the RXFP2 linker is shorter and lacks the region that is thought to interact with relaxin, the region (Region 1, Fig. 1A) involved in activation is highly conserved especially the key residues Asp43, Gly46, Trp47 and Phe51. To assess the role of region 1 in RXFP2 function we first mutated the residues individually to Alanine in RXFP2 and expressed them in HEK-293T cells to assess the effects on INSL3 and H2 relaxin binding and activation.

Binding studies with Eu-INSL3 demonstrated that mutations of linker residues did not affect INSL3 binding. However, activation studies highlighted that the linker is essential for INSL3-mediated activation, in particular residues Asp43, Gly46, Trp47 and Phe51. The key role of these linker residues in INSL3 mediated RXFP2 activation mirrors the role of the same residues in relaxin mediated RXFP1 activation^27^. However, in the case of RXFP1, mutation of region 1 residues also disrupts the structure of the binding site in region 2 resulting in loss of relaxin binding.

As anticipated, activation by H2 relaxin was also disrupted in the RXFP2 linker mutants. However, somewhat unexpectedly binding studies using Eu-H2 relaxin demonstrated that linker mutations disrupted H2 relaxin binding. It is therefore evident that although the RXFP1 region 2 is not present in the RXFP2 linker that H2 relaxin still has some binding interaction which is disrupted by the mutation of linker region 1. To assess the potential regions of the LDLa module and linker that may be involved in this interaction we utilized a similar approach to that which was used to determine the H2 relaxin-linker interaction site. Hence we designed an RXFP2 LDLa-linker protein construct to study the interactions with H2 relaxin using solution NMR.

The NMR titration studies for the RXFP2 linker interaction with truncated relaxin highlighted a specific interaction which involved the C-terminus of the LDLa module along with the first 8 residues of linker (Gly42 to Ile50) (Fig. 5). The involvement of calcium-binding residues Asp30 and Glu38 reflects the functioning of several LDLa modules from other proteins, which often bind ligand via the acidic residues that also bind calcium^42–44^. Testing the potential role of calcium-binding residues in RXFP2 by site-directed mutagenesis is problematic however, as it tends to interfere with correct folding of the module, and hence renders the receptors non-functional^35, 45, 46^. However, mutagenesis on the N-terminal residues of the linker support the notion that there is a weak relaxin interaction within this region (GDxxGWxxxF), contrary to what was reported for RXFP1.

**Figure 7:**
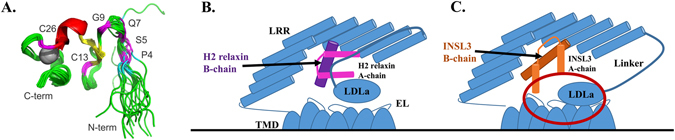
(**A**) Structure of the RXFP2 LDLa module (pdb: 2m96) showing the mainchain positions of Pro4 in cyan Ser5, Gln7, Gly9, Cys13 and Cys26 in magenta. The Ca^2+^ is represented as a grey sphere. (**B**,**C**) Diagrammatic representation of the extracellular portion of RXFP2 (blue) demonstrating proposed mechanism of activation in response to H2 relaxin (pink) and INSL3 (orange). The B-chain of the two peptides (labelled and in darker colour) sit at different orientations to one another, with relaxin at a 45° angle across a more restricted portion of the LRRs than INSL3, which requires more contacts and sits perpendicular to the LRR concave surface^41^. While the final active conformation of receptor in response to relaxin is driven primarily by the LDLa module, the INSL3 response also involves key residues in the N-terminus of the A-chain (circled in red).

Currently, an absence of ^1^H-^1^H NOEs from the linker prevents characterization of structure for either the LDLa-linker of RXFP1 or RXFP2. However, the apparent splitting of the indole NH signal of Trp47 by cis-trans isomerism of Pro4 of RXFP2_(1–65)_ as well as the observed heterogeneity in glycine resonances, suggests that the linker residues C-terminal to the LDLa module (GDxxGWxxxF) may in fact fold around to come into close contact with the N-terminus of the receptor. The structures of the LDLa module of RXFP1 (pdb: 2jm4)^35^ and RXFP2 (pdb: 2m96)^47^ have been solved in the absence of the linker. However, inspection of the structures, especially the LDLa module of RXFP2 (Fig. 7A) supports the possibility of the linker approaching the N-terminus. Currently, in these models the Cα of Pro4 and Gly42, the C-terminal residue, are 20 Å apart. Addition of a roughly extended structure of six residues, thus including Trp47, would place this latter residue within 5 Å of Pro4. (Fig.7A).

The role of the RXFP1 linker was previously shown to also involve an interaction with EL2 of the receptor TMD. A titration of the recombinant protein RXFP1_(1–72)_, consisting of the LDLa module and the 32-residue linker, with a soluble scaffold displaying partial EL1 and full-length EL2 revealed a potential binding site between residues within or near the GDxxGW motif and Phe564 and Pro565 of EL2^27^. In order to explore whether the same motif in the RXFP2 linker was also interacting with the extracellular loops a titration was performed with RXFP2_(1–65)_-P4F and the same RXFP1 soluble scaffold protein displaying EL2 and a shortened EL1 (ssRXFP1) as well as an equivalent RXFP2 scaffold protein (ssRXFP2). As the LDLa modules can be interchanged without a dramatic loss of signal we expected RXFP2_(1–65)_–P4F to interact with both scaffolds^36^. Indeed, titrations with RXFP2_(1–65)_-P4F showed interactions with the scaffold constructs, displaying two potential interaction sites, one clustered around the N terminus of the LDLa module and the other within the linker (Gly42 –His55) (Fig. 6). Residues that showed the greatest degree of chemical shift change are in the N-terminal region (Phe4 to Gly9), Cys13 and Cys26 (Fig. 6). Cys13 is near the N-terminal region while Cys26 appears distant (Fig. 7A). Interestingly, Cys26 also underwent significant chemical shift change in the relaxin titration. This residue resides close to the C terminus of the LDLa module and thus to the N terminus of the linker region (Gly42 to His55), suggesting that movement of this residue might be indicative of a fold change within the LDLa module upon ligand binding. Weak shifts are observed within the GDxxGWxxxF motif in both titrations (Fig. 6) which is consistent with the N- and C-terminals of the LDLa module approaching each other, explaining the large shift of Cys26. Nevertheless, small shifts are observed throughout the module, especially for the titration of the ssRXFP1 scaffold (Fig. 6), perhaps suggesting multiple binding modes. Consistent with this idea, we found that while the LDLa module was essential for activation of RXFP2, in contrast to RXFP1, site-directed mutagenesis did not identify specific residues of the LDLa module involved in receptor activation^47^.

The data from our study are consistent with previous studies on relaxin and INSL3 peptide analogues and RXFP1/2 chimeric receptors. Replacement of either the INSL3 A-chain or the B-chain individually with that of relaxin gives rise to poor activators of RXFP2^48^. Additionally, truncation of the relaxin A-chain leads to peptides that have concurrent drops in both binding and activation of RXFP1 and RXFP2^49^, whereas similar truncations on INSL3 maintain RXFP2 binding but lose their ability to signal^50^. Furthermore, a truncated INSL3 that is missing the first 9 residues of the A-chain acts as an antagonist to INSL3 activity^50^, as do some B-chain only variants^51^. The different roles of the peptide A-chains are consistent with our previous study where we have demonstrated that the peptide B-chains have distinct binding orientations to the LRRs of RXFP1 and RXFP2 respectively^41^. Relaxin interacts with RXFP1 by binding of the relaxin B chain α-helix across the face of the LRR at an angle of 45° whereas the binding mode of INSL3 to the RXFP2 LRRs requires a 90° binding angle between the LRR β-strands and the B chain α-helix. This study also highlighted that relaxin is likely to bind to RXFP2 in a manner similar to the way it binds to RXFP1.

These previous results and data from our current study lead to a proposed model of activation of RXFP2 by relaxin and INSL3 (Fig. 7B,C). For the relaxin-RXFP1 interaction binding to the LRR and linker directs LDLa-linker mediated activation whereas for INSL3-RXFP2 binding to the LRRs perhaps directs linker mediated reorientation of the LDLa module to activate the TMD in conjunction with the N-terminus of the INSL3 A-chain (Fig. 7C). In contrast, relaxin appears to activate RXFP2 in an RXFP1-like manner (Fig. 7B). Relaxin could therefore bind to the GDxxGWxxxF motif and reorient the N-terminus of the LDLa module to bind to the exoloops of the TMD, in a similar, yet novel and different mechanism of RXFP2 activation.

## Methods

### Receptor expression in HEK293T cells

Receptors were assayed after transient transfection into human embryonic kidney 293 T (HEK293T) cells (ATCC #CRL-1573; American Type Tissue Culture Collection) using lipofectAMINE 2000 (Invitrogen) per manufacturer’s instructions. Cells were grown in Dulbecco’s modified Eagle medium supplemented with 10% fetal bovine serum, 1% l-glutamine and 1% penicillin/streptomycin (referred to as complete DMEM) in 37 °C incubators with 5% CO_2_ and 85% humidity.

### Cloning and site-directed mutagenesis

Wild-type RXFP2 cDNA with a bovine prolactin signal sequence was inserted in a pcDNA3.1^TM^/Zeo + AmpR expression vector (Thermo Fisher Scientific) using BamHI and XhoI restriction sites. GB1-RXFP2_(1–65)_ and ssRXFP1 were both inserted into pET15b expression vectors (Novagen Inc.) using NcoI and BamHI restriction sites and NdeI and PstI restriction sites respectively. ssRXFP2 was inserted into a pET28a vector (Novagen Inc.) between NcoI and BamHI sites. GB1-RXFP2_(1–65)_ and ssRXFP2 were produced commercially at Genscript, NJ, USA. Single point-mutations for all mutants except RXFP2-G42A, RXFP2-G46A and RXFP2-F51A were introduced into wild-type receptor using PrimeSTAR DNA *Taq* polymerase (Takara Clontech) according to manufacturer’s instructions. Forward and reverse strands for mutants G42A and G46A were amplified separately and resulting single-stranded plasmids were annealed together in a further reaction (1 min at 95 °C followed by cooling to room temperature). RXFP2-F51A was made according to the protocol outlined in^52^. Mutant Primers are in Supplementary Table 1. Following PCR, 20 µl of a 50 µl total reaction mixture was added to 1 µl of DpnI (Promega) for approximately 2 hours before transformation into competent DH5α or top10 *E. coli* cells for subsequent DNA extraction using a Bioline Isolate II or a Promega Wizard Plus SV miniprep kit. All mutations were verified by sequencing and entire inserts were sequenced to ensure no accidental mutations occurred during the process.

### Eu3+- labelled INSL3- and H2 relaxin-binding assay

Comparison of ligand affinity for mutant receptors with wild-type RXFP2 was assessed using Europium (Eu^3+^)-labelled INSL3 (Eu-INSL3)^53^ or H2 relaxin (Eu-H2)^54^ at increasing concentrations in the presence or absence of 1 µM unlabeled ligand. Following a 1 h incubation, media was removed and 100 µl Delfia Enhancement solution (PerkinElmer) was added to each well. Plates were incubated in low light for 20–30 mins with shaking and then read on an Omega POLARstar plate reader using a time-resolved fluorescence protocol with excitation at 340 nm and emission at 614 nm. Data from at least three independent experiments, all performed in triplicate were pooled and presented as mean fluorescent specific binding ± SEM using GraphPad PRISM 6. Experiments were further analyzed by nonlinear regression one-site binding curves and *K*_d_ values were subjected to one-way analysis of variance (ANOVA) and uncorrected Fisher’s least square difference comparison test.

### cAMP activity assay

cAMP activation in response to ligand stimulation in HEK293T cells was measured using a colorimetric assay^55^ involving a pCRE β-galactosidase reporter gene that was co-transfected with receptors and empty pcDNA3.1^TM^ vector at a 2:1:5 ratio. Transfected cells were incubated at 37 °C for 18 hours before being stimulated with increasing concentrations of H2 relaxin or INSL3 prepared in complete DMEM. Positive and negative controls were done using 5 µM Forskolin or complete DMEM respectively. Following incubations for 6 h at 37 °C, media was aspirated and plates frozen at −80 °C for ≥ 24 h. Development of plates was achieved as previously described^27^ and readings were taken on a Benchmark Plus Microplate Reader (Bio-Rad) at 570 nm. All experiments were performed in triplicate a minimum of three times and data were pooled and presented as percentages of the maximum response induced by wild-type RXFP2 ± SEM. GraphPad PRISM was used to fit a nonlinear regression sigmoidal dose-response curve and resulting pEC_50_ and maximum response (*E*_max_) values were subjected to one-way ANOVA and uncorrected Fisher’s least square difference comparison test using only wild-type RXFP2 that was on the same plate as any particular mutant for comparison.

### Expression and purification of RXFP2_(1–65)_

RXFP2_(1–65)_ was designed with a GB1 solubility tag on both the N- and C-termini as well as an N-terminal His_6_ tag which was removable along with the N-terminal GB1 by thrombin cleavage. The construct was expressed in BL21 (DE3) *trxB* (Novagen) cells using autoinduction^56^. N5052 medium was used for uniform ^15^N-labelling using ^15^NH_4_Cl (Sigma-Aldrich)^56^ and the method described in^57^ was employed for ^13^C, ^15^N labelling, with cells grown in a 1 L Braun Biostat fermenter, and ^15^NH_4_Cl and D-[^13^C] glucose being the only nitrogen and carbon sources. Following harvesting and pelleting cells were stored at −20 °C.

Cells were resuspended in 20 mM Tris-HCl (pH 7.4), 150 mM NaCl, 5 mM Imidazole and lysed with an Avestin EmulsiFlex C3 cell crusher. The soluble fraction was separated from insoluble debris by centrifugation at 4 °C, 13,000 × *g* for 40 mins and then loaded onto approximately 10 ml pre-equilibrated (with 20 mM Tris-HCl (pH 7.4), 150 mM NaCl, 5 mM imidazole) Talon Superflow resin (Takara Clontech) to be purified by affinity chromatography. Elution was done with 400 mM imidazole in 20 mM Tris-HCl (pH 7.4), 150 mM NaCl and the resulting protein refolded overnight in a mixed redox reaction with stirring (3 mM GSH, 0.3 mM GSSG, 50 mM Tris-HCl (pH 8.5), 150 mM NaCl, 10 mM CaCl_2_), such that the three disulfide bonds of the LDLa module should form correctly. The His_6_-GB1 tags were removed by overnight incubation with 5 units of thrombin (Sigma-Aldrich) per mg of protein, and a second pass over Talon Superflow resin. Flowthrough and wash fractions were collected and further purified by reversed-phase high-performance liquid chromatography (RP-HPLC) (buffer A 0.1% trifluoro-acetic acid, buffer B 100% acetonitrile with 0.1% trifluoro-acetic acid) using an Agilent Zorbax 300SB-C18 column. Purity and molecular weight of collected fractions were assessed by mass spectrometry before they were lyophilized and stored at −20 °C.

### Expression and purification of ssRXFP1 and ssRXFP2

The scaffold proteins were expressed in BL21 (DE3) cells and purified as described for the RXFP1 scaffold protein previously^27^. Briefly, after 16 h of expression at 16 °C via isopropyl –D-1-thiogalactopyranoside (IPTG) induction in LB medium after cells reached an OD_600_ of 0.6, cells were harvested, pelleted and purified by affinity chromatography over Talon Superflow resin (Takara Clontech). The His_6_ affinity tag was removed by thrombin cleavage and the proteins further purified with a HiLoad 16/60 Superdex 75 prep grade column (GE Healthcare) in 20 mM Tris HCl (pH 7.4), 150 mM NaCl. Purity and molecular mass was assessed by polyacrylamide gel electrophoresis and mass spectrometry.

### Circular dichroism (CD) analysis of ssRXFP2

The ssRXFP2 construct had not previously undergone characterization or quality control, so it was submitted to CD measurements to ensure secondary structure was present and refolding had occurred. An AVIV Model 410SF spectropolarimeter (Biomed) was used and samples at 0.15 mg/ml were placed in a cell with a 1 mm path length for wavelength scans to be performed from 260–190 nm at 1 nm intervals. Buffer used was 20 mM Tris HCl (pH 7.4), 100 mM NaCl. Background (buffer only) readings were subtracted and data were expressed as mean residue elipticity (MRE, [θ]) according to the equation [θ] = (θ_obs_)/(l.c.n/M) where θ_obs_ = observed elipticity (mdeg), l = path length (cm), n = number of amino acid residues, M = the molecular weight and c* = *concentration (mg/mL). Data were analyzed using the online Dichroweb server with the Selcon3 method and reference dataset 7^58^, and compared to data previously acquired for the tGB1 protein for estimates of expected structure^24^.

### Production of B5-29 [B-K 9 R,A-K9/17 R] (truncated) H2 relaxin

A B-chain truncated analog of H2 relaxin with lysine residues at position 9 on the B-chain and positions 9 and 17 on the A-chain replaced by arginine (B5-29 [B-K9R,A-K9/17 R] H2 relaxin, referred to as truncated relaxin) was produced in *Pichia pastoris* as previously reported for [B-K9R,A-K9/17 R] H2 relaxin^32^. The expression construct for [B-K9R,A-K9/17 R] H2 relaxin was produced by site-directed mutagenesis using QuickChange methodology using the expression construct pPinkα-HC/[B-K9R,A-K9/17 R] H2 relaxin as the template^32^. Thereafter, the mutant H2 relaxin precursor was overexpressed in *Pichia pastoris*, purified from the culture broth, enzymatically treated to produce the mature peptide and purified by HPLC as described previously^32, 59^. Briefly, the purified single-chain precursor was treated with cyanogen bromide (CNBr) and the N-terminally shortened precursor purified by high performance liquid chromatography (HPLC) using a C18 reverse-phase column (Zorbax 300SB-C18, 9.4 × 250 mm; Agilent Technologies, Santa Clara, CA, USA). Finally, the shortened precursor was sequentially treated with endoproteinase Lys-C, papaya glutaminyl cyclase, and carboxypeptidase B and the mature two-chain relaxin purified HPLC using a C18 reverse-phase column (Zorbax 300SB-C18, 9.4 × 250 mm; Agilent Technologies). After lyophilisation, its purity and molecular mass was confirmed by mass spectrometry. The peptide was tested for its activity at both RXFP1 and RXFP2 in HEK-293T cells overexpressing the receptors and a pCRE reporter gene as described above^23^.

### NMR Spectroscopy

NMR experiments were all performed at 25 °C on a 700-MHz Bruker Avance HDIII spectrometer equipped with triple resonance cryoprobe, with proteins suspended in 50 mM imidazole, 10 mM CaCl_2_ at pH 6.8. Proteins for titrations were dialyzed in the same buffer and same vessel overnight. Backbone resonances (^13^Cα, ^13^Cβ, ^13^C’, ^15^N and NH) of residues were assigned from 3D HNCACB, HN(CO)CACB, HNCO and HN(CA)CO experiments using non-uniform sampling (NUS) on a Bruker Avance II HD 800 MHz spectrometer equipped with TCI cryoprobe at 298 K. For NUS, sampling schedules were generated using poisson gap sampler with 10% of the total number of points collected for all the 3D NMR experiments^60^. Spectra were reconstructed with compressed sensing algorithm using qMDD^61^ and processed using NMRPipe^62^ as described in^27^ and data analyzed in SPARKY (Goddard, T.D. and Kneller, D.G., Univ. of California, San Francisco). Samples for titrations of truncated H2 relaxin, ssRXFP1 and ssRXFP2 to RXFP2_(1–65)_ were dialyzed in the same buffer as RXFP2_(1–65)_. Chemical shift and intensity changes were monitored via the acquisition of 2D ^1^H-^15^N Heteronuclear Single Quantum Coherence (HSQC; 2048 × 256 data points) as described for RXFP1_(1–72)_ in^27^, using increasing concentrations of each titrant against 50 µM RXFP2_(1–65)_. 2D ^1^H-^1^H NOESY and TOCSY of H2 relaxin and truncated relaxin were acquired on a 600-MHz Bruker Avance HDIII spectrometer at 25 °C. Samples were 500 µM, pH 6.8.

## Electronic supplementary material

Supplementary information
