# Supplementary material for: Distinct activation modes of the Relaxin Family Peptide Receptor 2 in response to insulin-like peptide 3 and relaxin
Source: Sci Rep. 2017 Jun 12;7:3294. doi: 10.1038/s41598-017-03638-4 (PMC5468325; doi:10.1038/s41598-017-03638-4)
Supplement: Supplementary file 1 — Supplementary information [file 41598_2017_3638_MOESM1_ESM.pdf]

Supplementary Information.

Distinct activation modes of the Relaxin Family Peptide Receptor 2 in response to insulin-like peptide 3 and relaxin

Shoni Bruell, Ashish Sethi, Nicholas Smith, Daniel J. Scott, Mohammed Akhter Hossain, Qing-Ping Wu, Zhan-Yun Guo, Emma J. Petrie, Paul R. Gooley, Ross A.D. Bathgate

Supplementary Information.

A. cAMP activation in RXFP1 cells

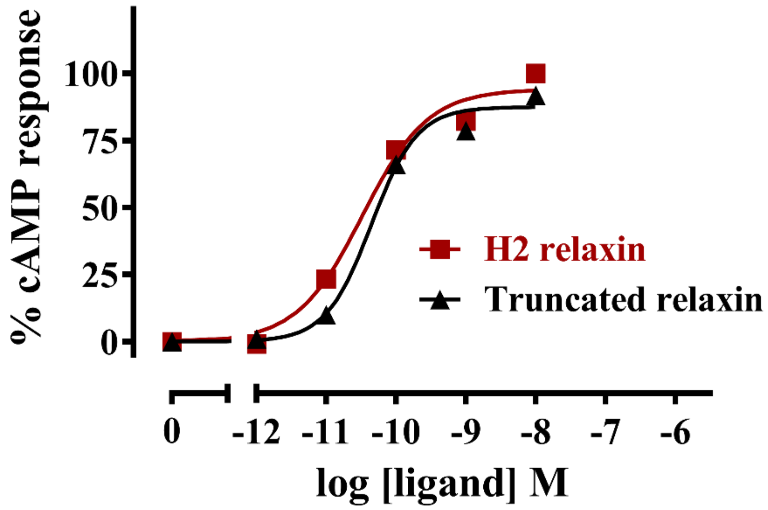

B. cAMP activation in RXFP2 cells

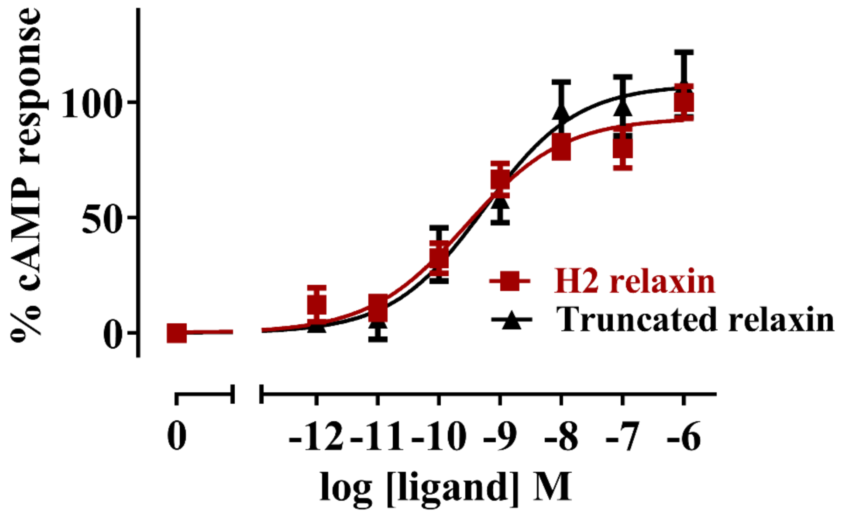

Supplementary Figure 1. cAMP response of **A.** stably transfected RXFP1 cells and **B.** transiently transfected RXFP2 cells to truncated relaxin in comparison to H2 relaxin. Data is presented as mean ± SEM of triplicate determinations from at least three independent experiments.

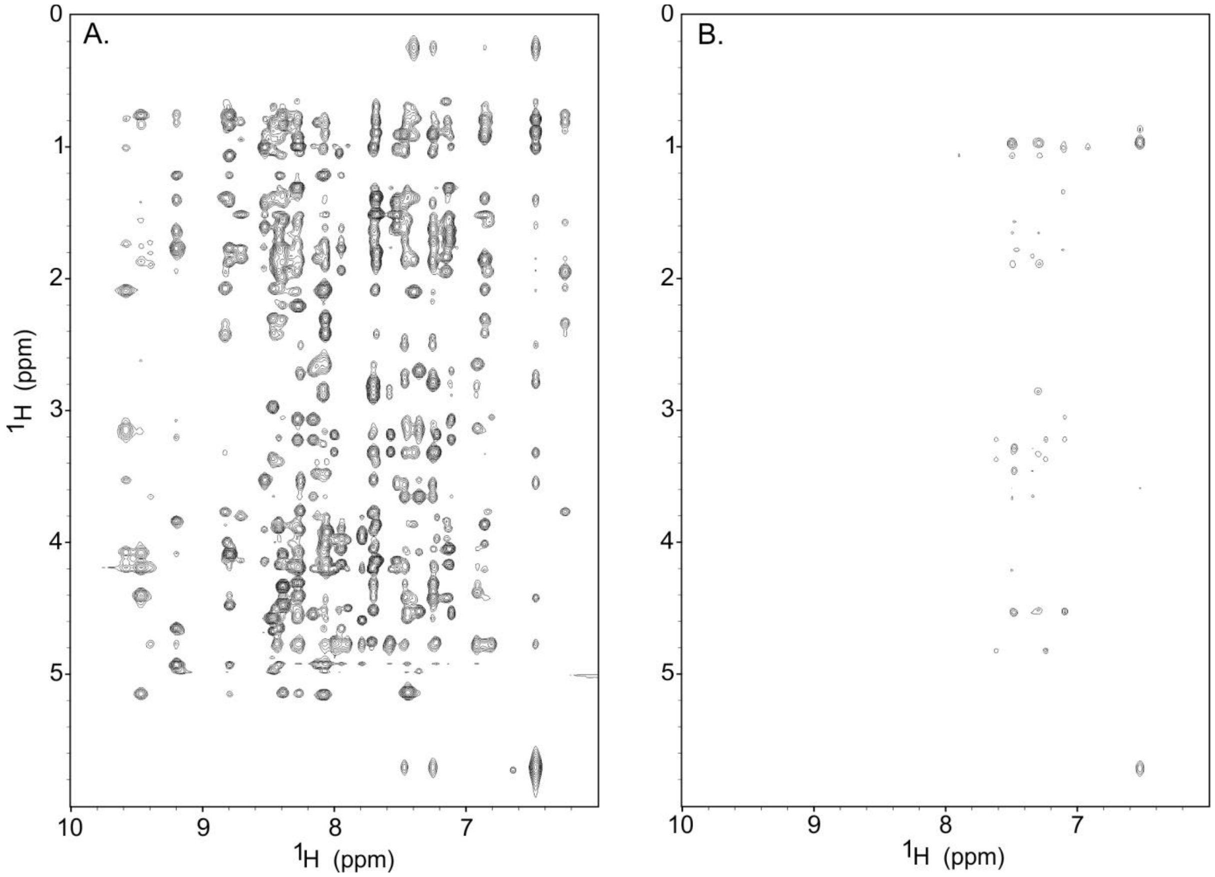

Supplementary Figure 2. 2D ^{1}H NOESY spectra of **A.** truncated and **B.** H2 relaxin at 500 µM.

A.

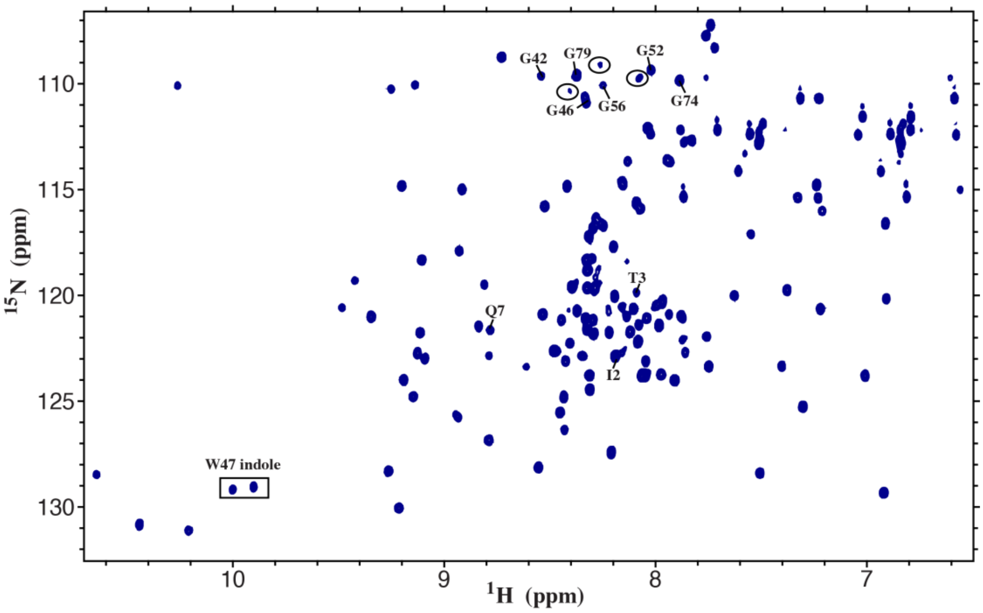

B.

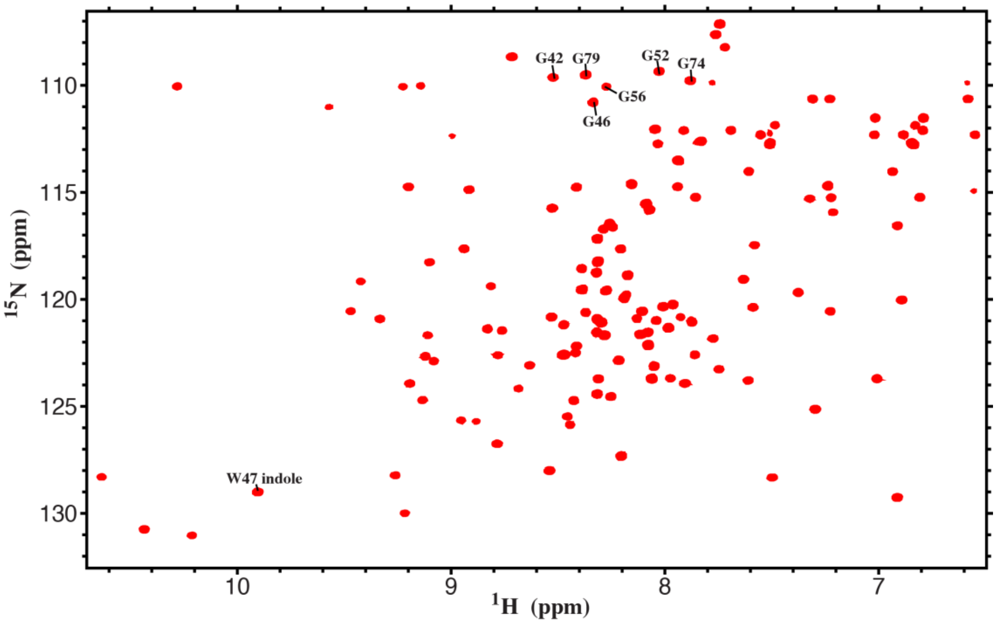

Supplementary Figure 3. Full 2D ^{1}H-^{15}N HSQC spectrum of **A.** RXFP2$_{(1-65)}$ and **B.** RXFP2$_{(1-65)}$ P4F highlighting the position and change in the indole signal of Trp47 upon mutation. Notably signals belonging to Glycines also show splitting

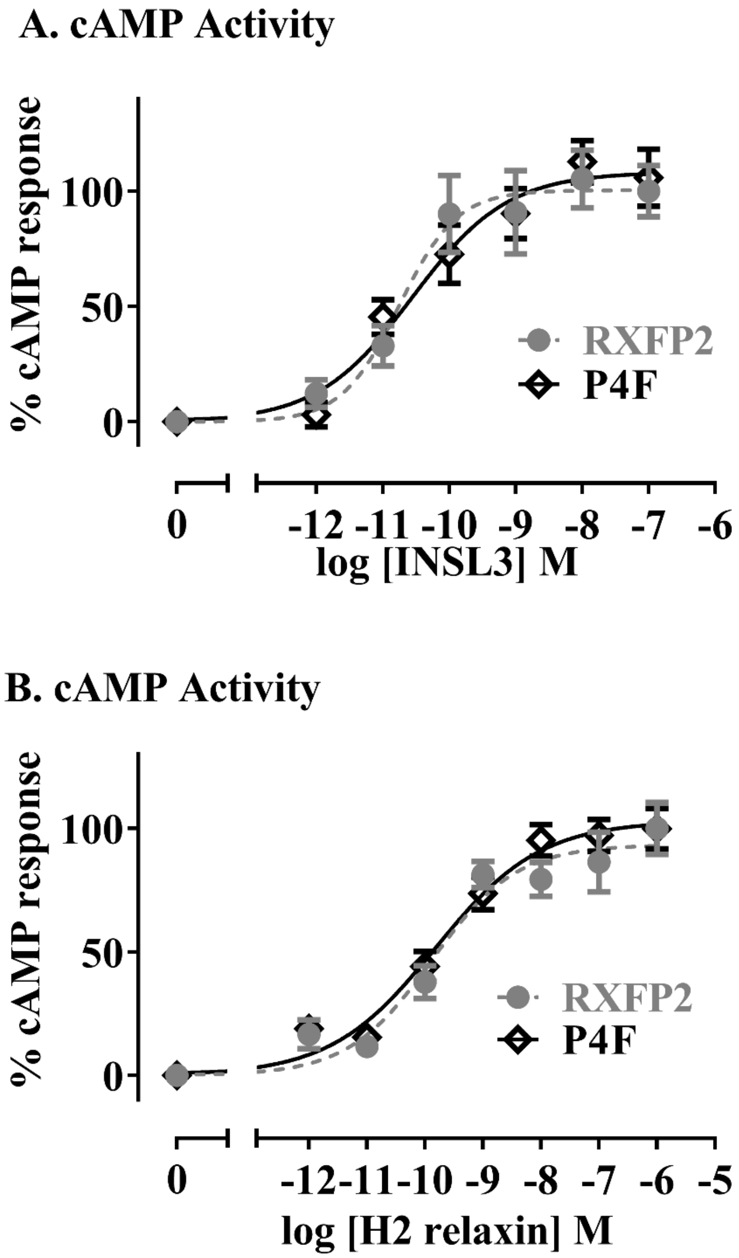

Supplementary Figure 4. A. INSL3- and **B.** H2 relaxin-induced cAMP response of RXFP2 mutant receptor P4F compared to wild-type RXFP2. Data is presented as mean ± SEM of triplicate determinations from at least three independent experiments.

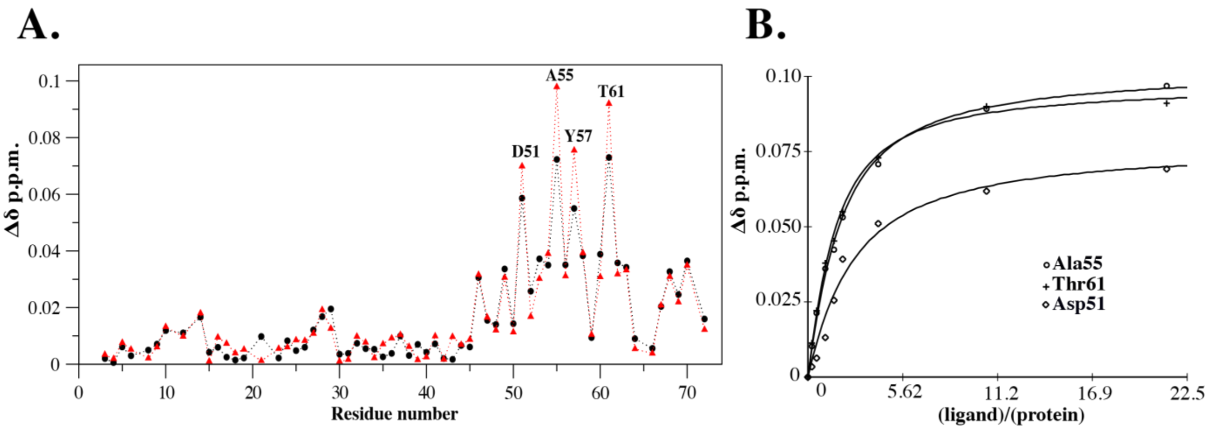

Supplementary Figure 5. A. Plot of the change in average ^{1}HN and ^{15}N chemical shifts following titration of ^{15}N-RXFP1$_{(1\text{–}72)}$ with 20 equivalents of H2 relaxin (black) or truncated relaxin (red). **B.** Single-site saturation binding curves (K_d = 90 ± 10 μM) for the resonances that show the largest chemical shift changes against truncated relaxin.

A. Sequence alignment of soluble scaffolds of RXFP1 and RXFP2 with tGB1

```
                      Thrombin cleavage site    Truncated EL1
ssRXFP1     MGSSHHHHHHSSGLVPRGSHMAQLWMESTHCQGGAQFKLIINGKTLKGEITIEAGG         56
ssRXFP2     MGSSHHHHHH---LVPRGSHMALLWMESVQCRGGAQFKLIINGKTLKGEITIEAGG         53
tGB1        -GSSHHHHHHSSGLVPRG--------------SGAQFKLIINGKTLKGEITIEAV-         37
                        EL2
ssRXFP1     EFFKNYYGTNGVCFPLHSEDTESIGAQGGAAEAEKIFKQYANDNGIDGEWTYDDATKTFTVTE  119
ssRXFP2     DYFGNFYGKNGVCFPLYYDQTEDIGSKGGAAEAEKIFKQYANDNGIDGEWTYDDATKTFTVTE  116
tGB1        ----------------------------DAAEAEKIFKQYANDNGIDGEWTYDDATKTFTVT-   75
```

B. SDS-PAGE gel of ssRXFP2 purification

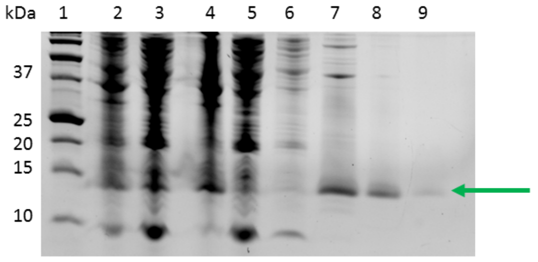

C. CD spectrum of ssRXFP2

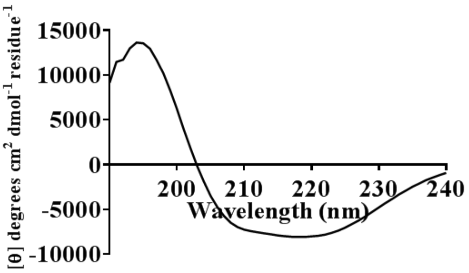

D. Estimation of structural content of ssRXFP2

Helix	Strand	Turns	Unordered
23.4%	34.5%	17.8%	21.8%

Supplementary Figure 6. Design and characterization of soluble scaffold protein ssRXFP2. **A.** Sequence alignment of ssRXFP1, ssRXFP2 and thermostabilised GB1 showing truncated EL1 (red) and EL2 (blue); **B.** SDS-PAGE gel of TALON purification of ssRXFP2 with (1) whole cells, (2) insoluble fraction, (3) soluble fraction, (4) flowthrough, (5) 5mM imidazole (imid) wash 1, (6) 5 mM imid wash 2, (7) 100 mM imid elution, (8) 200 mM imid elution, (9) 300 mM imid elution. Green arrow indicates position of HIS-ssRXFP2 (theoretical mol. wt. 12728.09 Da); **C.** CD spectrum of ssRXFP2 performed in 20 mM Tris (pH 7.4), 100 mM NaCl at 25 ° C; **D.** Estimated structural content of ssRXFP2 based on CD analysis.

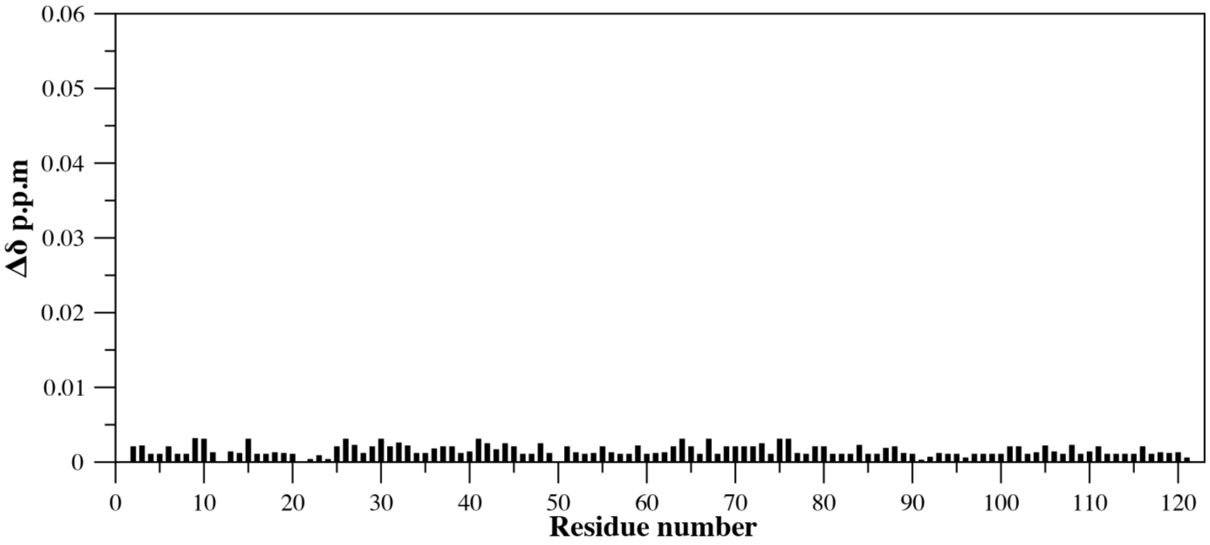

Supplementary Figure 7. Plot of the change in average ^{1}HN and ^{15}N chemical shifts following titration of ^{15}N-RXFP1$_{(1–65)}$ P4F with 20 equivalents of disulphide-less ssRXFP1.

Mutant	Sense primer (5'-3')	Antisense primer (5'-3')
RXFP2-P4F	CACTTTTTCATGCCAAAAAGGATATTTTCCCTGTGGG	CATGAAAAAGTGATCATGCTACCTTGGTCGACATC
$RXFP2_{(1-65)}$-P4F	GATCACCTTCAGCTGCCAAAAGGG	GGCAGCTGAAGGTGATCATGCTAC
G42A	CTGTGCTGACACTAGTGGATGGGCGACCATATTTG	GTGTCAGCACAGTTCTCTTCGTCCGCCCCG
D43A	GTGGTGCCACTAGTGGATGGGCGACCATATTTGGC	CTAGTGGCACCACAGTTCTCTTCGTCCGCCC
T44A	GTGACGCTAGTGGATGGGCGACCATATTTGGCAC	CACTAGCGTCACCACAGTTCTCTTCGTCCGC
S45A	CACTGCTGGATGGGCGACCATATTTGGCACAG	CATCCAGCAGTGTCACCACAGTTCTCTTCGTCCG
G46A	CTAGTGCATGGGCGACCATATTTGGCACAGTGC	CCCATGCACTAGTGTCACCACAGTTCTCTTCGTC
W47A	GTGGAGCGGCGACCATATTTGGCACAGTGCATGG	GTCGCCGCTCCACTAGTGTCACCACAGTTCTCTTC
F51A	CCATAGCTGGCACAGTGCATGGAAATGCTAACAGC	GTGCCAGCTATGGTCGCCCATCCACTAGTGTC

Supplementary Table 1. Mutagenic primers used in this study. Template was pcDNA3.1-RXFP2 (all) and pET28a-$RXFP2_{(1-65)}$ (P4F).
